# Are UK Policies and Practices for Regulated Donor Insemination Forcing Women to Find Unregulated Sperm Donors Online? A Perspective on the Available Evidence

**DOI:** 10.3389/fgwh.2022.644591

**Published:** 2022-02-21

**Authors:** Francesca Taylor, Rhys Turner-Moore, Allan Pacey, Georgina Louise Jones

**Affiliations:** ^1^Leeds School of Social Sciences, Leeds Beckett University, Leeds, United Kingdom; ^2^Department of Oncology and Metabolism, University of Sheffield, Sheffield, United Kingdom

**Keywords:** donor insemination (DI), reproductive justice (RJ), online sperm donation, fertility treatment, LGBTQ +, health inequalities, NHS funding

## Abstract

In recent years, there has been an increase in women obtaining donor sperm via unregulated websites and social media. In this article, we bring together the disparate evidence in this emerging field to consider whether restrictive UK policies and practices for regulated clinical donor insemination (DI) are a potential explanation for the growing use of the currently unregulated, online route to donor insemination. To this end, we examine the nature of the National Institute for Health and Care Excellence (NICE) guidelines, recent data provided by the Human Fertilisation and Embryology Authority (HFEA), and prior research on who uses online sperm donation and their reasons for doing so. In addition, we highlight why this issue is important by outlining some of the benefits and drawbacks of the unregulated route. We argue that, whilst there are many factors driving the unregulated route to DI, restrictive UK policies and practices for regulated DI might be one of these. We conclude that turning our attention to structural barriers, such as regulated DI policies and practices, is necessary to produce more definitive evidence of this potential issue, and that adopting a Reproductive Justice framework could lead to more equitable provision of regulated DI services.

## Introduction

In the United Kingdom (UK) and other countries with advanced healthcare systems, if an individual needs access to donor sperm to have a baby, they can do so via a regulated or unregulated route. In the UK, the regulated route is via the HFEA licensed clinics (1). The HFEA began regulating sperm donation in the UK on 1st August 1991. They regulate all fertility treatment by setting standards, licensing and providing guidance on how clinics and other projects involving human embryos can meet the legal requirements set out in the Human Fertilisation and Embryology (HFE) Acts (1990, 2008) through their Code of Practice (2, 3). A key requirement of the HFE Act 1990 was that sperm donors were legally protected, and infertile males could be recognized as the legal father of any children born through sperm donation (4). During the time that the HFE Act 1990 was being debated, the British media covered a high-profile case of a 40-year-old lesbian woman who was receiving treatment with donor sperm in the UK (5–7). Steinberg has since argued that the media's framing of the woman's desire to have a child as “selfish and deviant” was an influencing factor in the subsequent creation of a clause which restricted treatment to those with male partners, known as the “need for a father” (8, 9). Despite the controversial 2008 amendment of the “need for a father” clause to the “need for supportive parenting”- which recognizes married or civilly partnered same-sex partners as the legal parents of children conceived through sperm donation- the early heteronormative framing of regulated fertility treatment has arguably had lasting effects on HFEA practices and guidelines for treatment, which we explore further in this article (9, 10).

The unregulated route to sperm donation involves obtaining sperm from a known donor (friend or family member), or from a donor met on a “connection” website or social media platform for self-insemination at home. There is evidence to suggest that unregulated sperm donation - also termed “private,” “known,” “informal” and “self-arranged” donation - has been practiced, primarily by same-sex female couples, since the 1970s (11). Sperm was obtained from a friend or family member, or via “self-insemination networks” (a group formed outside of medical structures, comprising anonymous and/or known donors and recipients) (12). In recent years, there has been an increase in individuals obtaining donor sperm online. While exact numbers on the size of the online sperm donation market are unknown, the preliminary findings of our environmental scan of these sites estimates that there are more than 350,000 potential recipients on over 60 English-language websites and social media pages around the world. The environmental scan [see (13) for information on this method] involved systematically searching Google and Facebook for websites and social media groups which facilitate contact between donors and recipients, and then recording the membership figures of these sites/groups. The full results from this study are yet to be published.

In this perspective article, we consider whether the UK policies and practices for regulated DI are potential reasons for individuals sourcing sperm online. In so doing, we bring together the disparate evidence in this emerging field: the nature of the NICE Guidelines for fertility treatment and recent data from the HFEA pertaining to regulated DI; and prior research concerning online, unregulated sperm donation, focusing on who is choosing this route and their reasons for doing so. On the basis of the available evidence, we argue that “stratified reproduction”- which sees less wealthy and more marginalized groups having reduced access to regulated treatment- is becoming increasingly apparent in the context of regulated donor insemination in the UK (14, 15). We propose that, as a consequence, increasing numbers of single women and women in same-sex couples from low-income households and proportionally more from ethnic minority backgrounds, are looking for unregulated sperm donors online. We highlight why this issue is deserving of attention by outlining some of the benefits and drawbacks of the online route, concluding by proposing potential solutions to address the inequity in provision of regulated DI services in the UK.

## Policies and Practices in Regulated Donor Insemination

**Figure 1:**
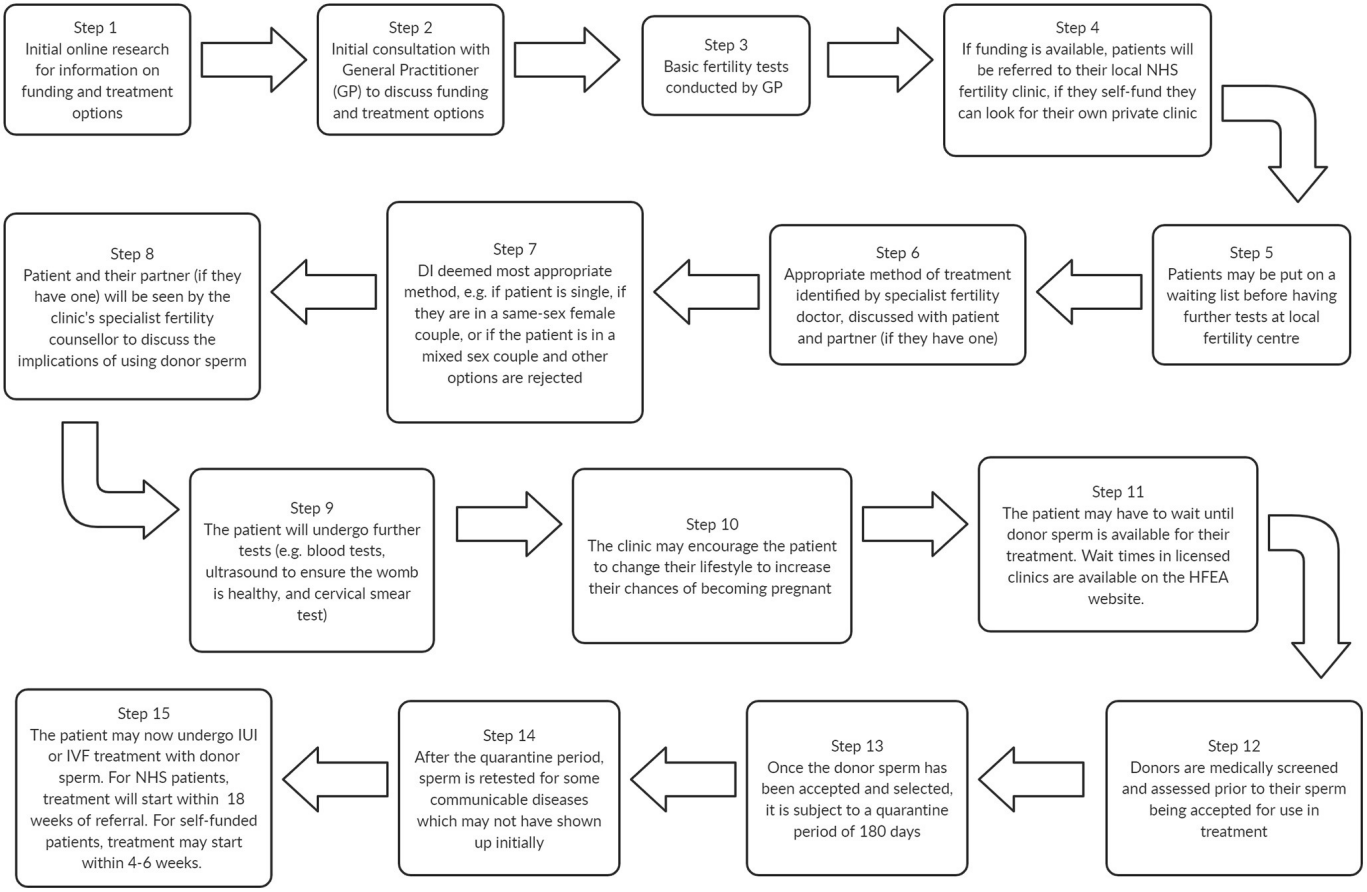
A typical donor insemination journey at a licensed clinic prior to receiving treatment, adapted from (16, 17).

The typical journey for a patient undergoing Donor Insemination (DI) at a HFEA-regulated clinic is depicted in Figure 1. In a regulated setting, sperm is procured from donors recruited by the clinic's “in-house” bank (if they have one), or from a commercial sperm bank in the UK or abroad. Once the sperm has been selected and the recipient is ready for treatment, they may then undergo either Intrauterine Insemination (IUI) or *In-vitro* Fertilisation (IVF) (see Table 1).

**Table 1:** NICE guidelines on assisted reproductive technologies (ART) using donor sperm (16).

Method	Intracervical Insemination (ICI). A procedure in which sperm is placed inside a woman's cervix to help her conceive. This method is rarely used at regulated clinics due to IUI having higher success rates.	Intrauterine Insemination (IUI). A procedure in which sperm is placed into a woman's womb to help her conceive. This method has higher chances of conception than ICI, even if the sperm has been frozen and thawed. IUI should be unstimulated in the first instance but can be stimulated (using fertility medicine) if a fertility problem has been diagnosed.	*In vitro* Fertilisation (IVF). This treatment begins with stimulation of the ovaries through use of fertility medicine. Eggs and sperm are then collected and fertilized outside the body. One or two embryos are then selected and placed into the womb. This procedure can be used in combination with Intracytoplasmic Sperm Injection (ICSI) in severe cases of male infertility.
Donor or partner sperm	Either (only if partner sperm has to be washed)	Either	Either
Sperm type	•Fresh and unwashed (higher chances than frozen and thawed) • Fresh and washed (for those with a viral infection that can be sexually transmitted) • Frozen and thawed (e.g., imported from a sperm bank)	•Fresh and washed • Frozen and thawed (e.g., obtained from a sperm bank)	•Fresh and washed • Frozen and thawed (e.g., obtained from a sperm bank)
Setting	•Self-insemination at home (unregulated route) • In a licensed clinic if sperm is being washed due to a viral infection	In a licensed clinic	In a licensed clinic
Suitable for	•Single women using donor sperm with no infertility diagnosis • Same sex couples using donor sperm with no infertility diagnosis • Mixed sex couples where the sperm-producing partner has a viral infection that can be sexually transmitted	•Single women using donor sperm • Same sex couples using donor sperm • Mixed sex couples using partner or donor sperm	•Single women using donor sperm • Same sex couples using donor sperm • Mixed sex couples using donor or partner sperm
NHS funding example (dependent on CCG)	ICI is not routinely funded by the NHS	•Same sex couples who have not become pregnant after 6 self-funded cycles of “artificial insemination” (not specified) • Mixed sex couples where the sperm-producing partner has a viral infection that can be sexually transmitted and so sperm must be washed (if ICI not available at clinic) • Mixed sex couples who are unable to have sexual intercourse (e.g., because of a physical disability or psychosexual disorder)	•Same sex couples who have not conceived after 12 cycles of artificial insemination (where 6 or more are by IUI) • Mixed sex couples who have not conceived after 2 years of regular unprotected intercourse or 12 cycles of artificial insemination (where six or more are by IUI)

In England, NICE provides guidelines for healthcare professionals on diagnosing and treating all health—including fertility—problems. This involves setting out the criteria on who should be offered what treatments and why (18). Table 1 displays the NICE guidelines on Assistive Reproductive Technologies (ARTs) using donor sperm, which are used by General Practitioners and fertility specialists at HFEA licensed clinics. The guidelines suggest that for mixed-sex couples to be eligible for NHS-funded treatment, they must have failed to conceive after regular unprotected intercourse for 2 years [depending on their local Clinical Commissioning Group (CCG)], although it is not clear how they provide proof of regularly trying for this time period. Despite same-sex couples not being able to conceive without assistance, NHS-funded treatment in England is only available to same-sex couples who first pay for six unsuccessful cycles of IUI with donor sperm (this number varies from 6 to 12, depending on the CCG) (19). For single women, it appears unlikely that they will be eligible for any NHS funding (19).

The cost of a single cycle of IUI at a private clinic varies from £800–1,600, plus the cost of donor sperm (~£1,000 for one vial), consultations and health tests (20). Evidently, same-sex couples from low-income households will struggle to self-fund six cycles of IUI in order to become eligible for NHS funding, while single women must be able to afford, or take out loans for, private treatment from the start and throughout their entire assisted conception journey. Additionally, as each local CCG can decide how to allocate their funds, the “postcode lottery” of regulated fertility treatment means that, even if same-sex couples become eligible for NHS-funded treatment after self-funding the initial six IUI cycles, their access to funded treatment will vary dramatically according to their location (21).

A comparison of the NICE criteria (Table 1) for mixed- and same-sex couples, and single women, illustrates inequity in the distribution of NHS resources, where rationing and policy decisions create stratified reproduction (14). Stratified reproduction is defined by Ginsburg and Rapp (22) as:

“The power relations by which some categories of people are empowered to nurture and reproduce, while others are disempowered... [and] arrangements by which some reproductive futures are valued while others are despised” (p. 3).

Other scholars from the UK and elsewhere have drawn attention to women's stratified access to ARTs based on divisions of sexuality, wealth, class and ethnicity (23, 24). The vast majority of research on regulated fertility treatment in industrialized countries has been conducted with white, middle-class, heterosexual women for whom ARTs are generally more available and/or affordable, leaving the experiences of minoritized women (e.g., LGBT+, poor, Black, Asian, and Indigenous) largely unexplored (24).

The HFEA report that in 2017, 5,603 DI cycles were performed via the regulated route (including both NHS and self-funded cycles) (25). There has been a slight upswing in overall regulated DI treatments in the UK, attributed to increases in same-sex couples and single women self-funding their fertility treatment, as well as Scotland's increase (from 22% in 2012 to 53% in 2017, up to 70% in 2018) in NHS-funded DI cycles (25, 26). The HFEA do not state why there has been an increase in same-sex couples and single women self-funding their treatment, but the lack of NHS funding for these groups is alluded to in the report (see below). The increase in NHS-funded DI cycles in Scotland has been attributed to increased NHS funding for all Scottish fertility services since 2012 (25, 27).

The state of funding in the rest of the UK tells a different story. In England, Wales and Northern Ireland there have been “dramatic changes” in NHS-funded DI cycles, which have been steadily decreasing since 2015 (25). In 2018, English mixed-sex couples received the highest levels of funded cycles (36% for IVF and 12% for DI), whilst there were lower levels of funding for female same-sex couples (11% for IVF and 3% for DI), and single women received the lowest level of funding overall (3% for IVF and 1% for DI) (26). Given the NICE guidelines, it is not clear why some (although very few) single women received funding. Compared with Scotland, which funded 61% of IVF and 47% of DI cycles for mixed-sex couples, 40% of IVF and 70% of DI cycles for female same-sex couples, and 28% of IVF and 16% of DI cycles for single women in 2018, England's figures are dismally low and consistently favor mixed-sex couples (26). The stringent NICE guidelines (Table 1), coupled with the localized CCG and Health Board criteria for DI, mean that fewer and fewer women—and same-sex couples and single women in particular—in England, Wales and Northern Ireland are eligible for NHS-funded treatment, that is, if DI is funded at all in their region (17, 25). The HFEA conclude that:

“Criteria for DI can mean people do not get treatment for DI under the NHS. This particularly impacts women in same-sex relationships or with no partner who do not necessarily have an infertility diagnosis, and more significantly, are unable to try to conceive naturally with their partner” (25, p. 32).

During the same period as the results from the aforementioned report were being finalized, in July 2019, the UK Health Secretary stated that “sexual orientation should not be a factor in access to IVF” and committed to carrying out a review into LGBT+ access to regulated fertility treatment (28). In response to the perceived unequal treatment of LGBT+ individuals seeking regulated fertility treatment in the UK, a petition has been set up which urges the government to “stop discriminating against LGBTQ+ families”, and to follow-up on its promise to conduct the review (29). The petition has gained over 30,000 signatories and the social media influencers who created it cite the “alternative and dangerous” online route that some of their followers are undertaking as a cheaper alternative (30). More recently, the British Pregnancy Advisory Service (BPAS) published the results of an investigation into NHS-funded fertility care across the UK for female same-sex couples, concluding that this group do indeed face “significant barriers” which have created a so-called tax on LGBT+ families wishing to access regulated fertility treatment (31).

A HFEA report, entitled “Ethnic diversity in fertility treatment 2018,” also published in 2021, highlights disparity in access to, and outcomes of, fertility treatment for ethnic minorities (32). From the HFEA data, and a comparison with statistics from the general population (also contained within the report), it is evident that white patients are overrepresented in regulated DI treatments (87% of UK population but 92% of DI treatments); Black patients are slightly underrepresented (3% of UK population but 2% of DI treatments), Asian patients are also underrepresented (7% of UK population but 3% of DI treatments) and those of mixed ethnicity are equally represented (2% of UK population and 2% of DI treatments) (32). When sexuality and ethnicity are considered together, even more disparity is evident, with 96% of same-sex couples receiving DI being white (32).

Unfortunately, the HFEA data does not provide a breakdown of NHS-funded vs. self-funded regulated DI treatments by sexuality and/or ethnicity so it is not clear how those at the intersection of these identities are impacted by a lack of state funding. It is, however, evident that individuals from minority ethnic communities are over-represented among low-income households and often reside in areas which have been hardest hit by sustained disinvestment in fertility treatment in England (24, 33). As a result of the evidence on increasing disparities for ethnic minorities, the HFEA are urging commissioners to “review their funding eligibility criteria to consider whether these have an adverse impact on access to treatment among particular ethnic groups” (32).

While access to funded DI is certainly one of the most important factors for individuals choosing which conception route to go by (23, 34), other aspects also inform this decision. For example, drawing on the literature pertaining to cross-border reproductive care, recipients have reported choosing overseas fertility treatment over treatment in the UK owing to: shorter waiting times, availability of donors, quicker test results, and lower costs (35). Indeed, the shortage of sperm donors registered with regulated clinics has been cited as a disadvantage of regulated DI in the UK, and can mean that patients must pay more to purchase and ship imported sperm from banks overseas (36). Furthermore, there is evidence to suggest that lesbian recipients report experiencing discrimination in regulated settings, including feeling as though they must justify their right to be parents, and having to repeatedly “come out” to various healthcare professionals (37, 38). Finally, although the evidence on the experiences of Black and minority ethnic individuals is distinctly lacking, we know that they experience poorer outcomes of, and face considerable challenges in accessing, regulated fertility treatment (39). In the US, reasons for such disparity have been attributed to a mistrust of healthcare systems due to previously experienced or perceived racism, and biased assumptions by health professionals about sexual behavior and the cause of infertility in Black women (39).

## Research on Unregulated Sperm Donation

**Figure 2:**
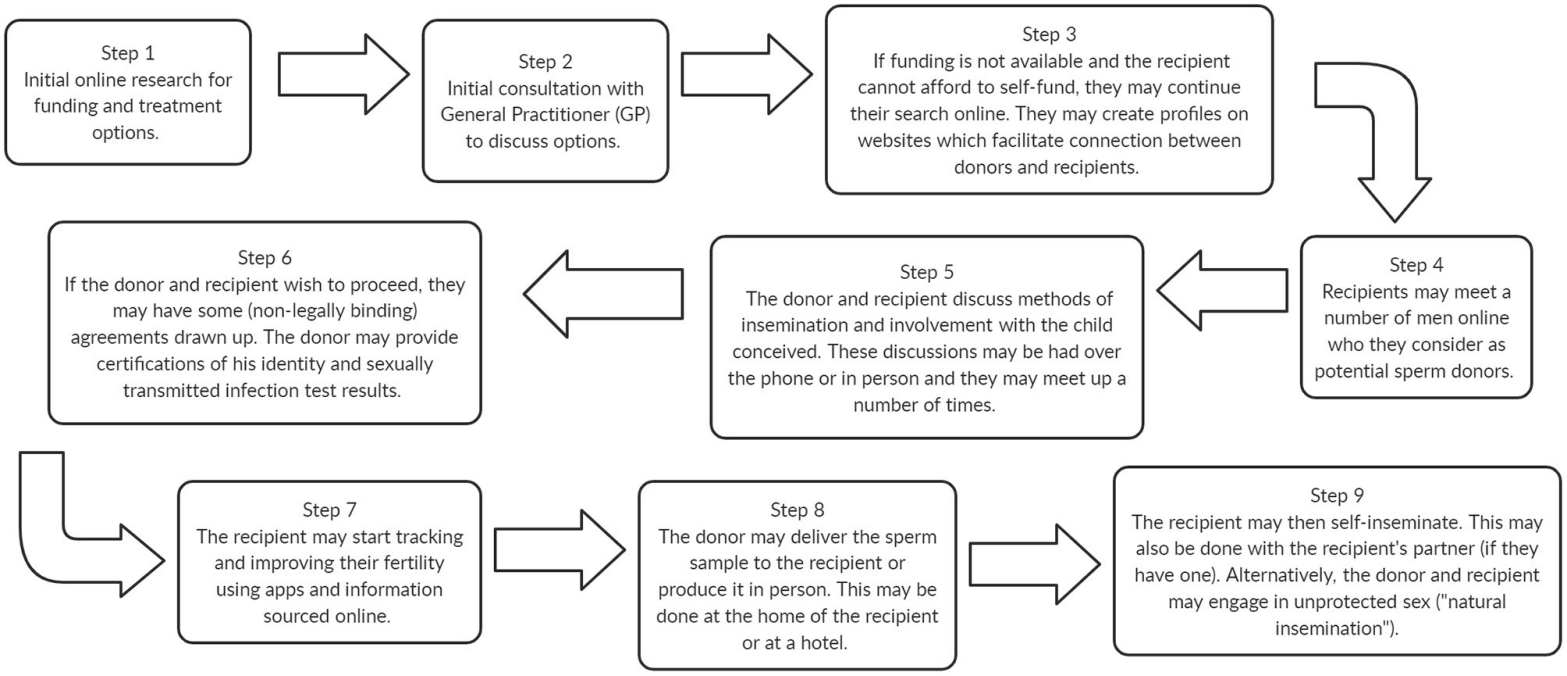
A typical unregulated, online donor insemination journey informed by our Patient and Public Involvement (PPI) group and (32).

At the same time as we have seen a reduction in funding for regulated DI treatment, there has been a significant increase in single women and women in same-sex couples obtaining donor sperm online (15, 40–42). The typical journey for a person obtaining donor sperm online is depicted in Figure 2. At-home insemination can be achieved via Intracervical Insemination (ICI; see Table 1) performed by the recipient or their partner, or through sexual acts with the donor. Sperm donation through online platforms falls outside of the HFEA's regulatory control as it takes place outside of a clinic.

Whilst advances in internet technologies and the habitual use of social media platforms are likely to have partly driven the increase in single women and women in same-sex couples obtaining donor sperm online, these are not the only drivers (40). As a relatively new phenomenon, there has been limited research into online sperm donation to date, and of this limited research, only two studies have explored the characteristics of recipients who are undertaking this route (41, 42), and only one has explored their motivations (41).

Both of these studies recruited participants from *Pride Angel*— one of the largest and most well-known connection websites—, which is UK-owned but used worldwide [sample sizes were *N* =429 and *N* =74, respectively (41, 42)]. The majority of participants in both studies were either British (58%, 25%), American (18%, 32%), or Australian (8%, 25%); LGBT+ (78%, 65%, compared to 2% of the UK population); and in a relationship (76%, 66%, compared to 50% of the UK population), with a sizeable minority of participants being single [24%, 34%, compared to 32% of the UK population (41–43)]. When compared with the aforementioned figures pertaining to NHS-funded DI cycles in regulated clinics, these findings suggest that women in same-sex female couples are underfunded by the NHS and proportionally over-represented in online sperm donation.

Furthermore, the majority of participants in both studies were white (84%, 91%); the other ethnicities in Whyte's study (42) are not recorded, but 6.5% of the participants in Jadva et al.'s study (41) were Black (compared to 3% of the UK population), followed by Asian (3%, compared to 7% of UK population), mixed race (4%, compared to 2% of the UK population) or another ethnicity (2%, the same as the UK population) (32). Again, these figures suggest that, while proportionally less Black and Asian patients are receiving DI treatment at regulated clinics, proportionally more individuals from Black and mixed-race populations are using online sperm donation.

Overall, the participant characteristics from the above studies suggest that LGBT+ women and those from Black and mixed minority ethnic backgrounds are proportionally more likely to use the online route. However, it is important to note that *Pride Angel* has a greater LGBT+ focus than some other “connection” websites or social media groups, which may present a misleading picture of how many sexual minorities use the online route. Furthermore, limited access to, or funding for, regulated DI services might not wholly explain the findings for online sperm donation. There is a lack of Black, Asian and mixed heritage donors in the regulated route. For example, 86% of sperm donors registered at UK clinics are white, which may lead to, for example, growing numbers of mixed heritage lesbian couples who are seeking donors who share their heritage, to turn to unregulated means of sperm donation, such as “connection” websites, or their own personal networks (32, 44).

Of the two studies, the Jadva et al. study (41) also surveyed recipients about their motivations for, and experiences of, searching for a sperm donor online, using multiple-choice and open-ended questions. Participants were asked to select their “main reason for seeking a sperm donor” from a predetermined list of options. The majority of recipients recorded their reason as “I am gay/lesbian” (64%), followed by “I do not have a partner to have children with” (10%). The list of options did not include “I cannot afford/did not qualify for funded treatment” (41). Over half of the sample (58%) reported that there were advantages to obtaining donated sperm via *Pride Angel*, and when asked to describe the advantages in their own words, these most commonly entailed: “being able to meet and connect with the donor” (24%), “fewer costs involved” (18%) and “availability of detailed information about donors” (13%) (41). Participants were also asked if they would consider obtaining donated sperm from other sources, including a sperm bank, fertility clinic, friend or other connection website. Of these, sperm bank (71%) and fertility clinic (63%) were most commonly selected (41). Overall, these findings suggest that recipients are aware that their sexuality and relationship status are factors influencing their decision to look for a donor and that “fewer costs” are an advantage of the connection website. Furthermore, given that the majority of participants would consider using a sperm bank or fertility clinic, but cite the “fewer costs” relating to the connection website, these findings allude to an awareness of the significant costs associated with private regulated treatment, suggesting that some participants had looked into the regulated route and found the costs prohibitive. However, it should also be recognized that increased information about the donor, and the opportunity to meet them, were also cited as benefits of the unregulated route.

Nevertheless, the limited evidence on recipients' motivations for taking the unregulated route is corroborated by (the also limited evidence on) unregulated donors. In two interview studies on their experiences of online sperm donation, unregulated sperm donors mentioned the prohibitive sociolegal context for marginalized groups of women as a motivation for donating (15, 45). For instance, one French donor described the recipients he donated to as “hard-working, non-affluent lesbian couples that were not able to access sperm through other channels due to unfavorable social and legal environments” [(15), p. 7]. In another study, a Canadian donor mentioned the substantial costs of regulated open-identity donation, which are not covered by state-funded programmes or private insurance, and the authors conclude that, “some single women or couples who want access to information about the donor turn to the Internet to meet this need” [(45), p. 8]. This demonstrates that motivations for online sperm donation are tied up with inaccessible regulated practices.

## The Benefits and Drawbacks of Unregulated Sperm Donation

As participants stated in the Jadva et al. (41) study in the previous section, online sperm donation, as well as being much cheaper than private regulated treatment, offers recipients the opportunity to meet up with and get to know their donor if they wish, and find out much more detailed information about the donor than that which is provided by clinics (41). The unregulated route is also much less medicalized, and some recipients would rather not unnecessarily involve clinics, doctors, nurses, appointments, medication and tests in their conception journey when they do not have a diagnosis of infertility (45). In this way, some recipients report that unregulated sperm donation enables them to have more control over the process: everything can be undertaken in the comfort of their own home, and their partner can be involved in the insemination process (37). It might be argued then, that the online sperm donation phenomenon has built upon the earlier known donation practices of the late 20th century and brought it into the globalized, online marketplace of today. These “DIY” approaches have been welcomed by those researching minority ethnic, LGBT+, and alternative families in the past, as they offer individuals the option of conceiving their children outside of the rigid medical structures and state regulation of ART, which arguably reinforce the aforementioned issues of stratified reproduction (46, 47).

However, while some recipients do prefer to conceive outside of regulated clinics, it is important to highlight that online, unregulated sperm donation is not for everyone, and individuals should be able to choose between the regulated and unregulated routes to conception. A number of scholars have started to explore the drawbacks of the unregulated route, highlighting some of the legal and health implications (37, 40, 48–50). Firstly, it has been noted that negotiating the legal issues of parenthood in unregulated arrangements can be complex. While regulated clinics are able to separate the donor legally from any offspring conceived, different rules apply if conception takes place outside of a clinic. In unregulated arrangements, the sperm donor is not the legal father if he donates by artificial insemination to a married or civilly partnered couple (51). However, if there is no second legal parent (e.g., the non-birth mother is in an unmarried couple, or the recipient is single), the sperm donor will be the legal father, irrespective of what the parents agree or what is recorded on the birth certificate (51). Further, a sperm donor who donates through sexual intercourse (sometimes called “natural insemination”) is always the legal father of any child conceived, irrespective of what the parents agree or what is recorded on the birth certificate (51). For unmarried couples, single women and anyone who conceives via sexual intercourse, then, there is the possibility that the donor could make a claim for legal parenthood, which could later be established in court (52). Whilst for donors, there can be concerns that recipients will “come after them” for child support (45).

There are also some health risks associated with unregulated sperm donation. For example, recipients must trust that donors are free from sexually transmitted infections (53). Although some sperm donation websites suggest that donors are tested and present their test results to recipients prior to insemination, there is no guarantee that they will not contract an infection between the tests being conducted and the donation being received (52). It is also difficult to safeguard against the risk of consanguinity and genetic illnesses in unregulated sperm donation arrangements (40). However, this issue is not unique to the unregulated route. There is evidence of donors in the US fathering hundreds of offspring through donations to different sperm banks and/or moving from clinic-based to online sperm donation (54). In some cases, sperm banks had failed to detect life-threatening genetic conditions in donors, which were inherited by donor offspring (54). In UK clinics, any one donor's sperm can be used to help a maximum of ten families (55); however, records extend only to HFEA licensed clinics. As there is no independent global register which records the identities and locations of the children each donor has fathered across HFEA licensed clinics, international sperm banks and the unregulated route, “Super Donors” (i.e., donors who purposefully have hundreds of biological offspring) exponentially increase the risk that half-siblings might unwittingly meet and have sexual relationships, and potentially children, with each other later down the line (56).

Lastly, there is anecdotal evidence in the media, and two studies to date, which indicate that women who use online sperm donation sites are being harassed and abused by donors (52, 57–60). McQuoid (52) undertook a “covert netnography”, assuming personas to gain access to the online sperm donation community over a period of 33 months, during which time she liaised directly with 198 female recipients, and 92 men. McQuoid found that half of the women (*n* = 99) involved in her research experienced abuse from online sperm donors, such as: physical, financial, emotional and verbal abuse; stalking; trolling; racism and homophobia; sexual grooming, harassment and rape. The author also draws attention to the abbreviations coined by unregulated donors for use on connection sites when discussing their preferred method of insemination (52). These abbreviations include: AI+, artificial insemination plus a sexual act performed by the recipient; PI, partial insertion of the penis into the recipient upon ejaculation, and Natural Insemination or NI, unprotected sex with the recipient (52). When this is combined with an insistence from some donors that the insemination success rate is higher if these sexual acts are performed, McQuoid argues that donors have “linguistically created superfluous culturally authentic ‘donation methods' to coerce or push women toward sexual intercourse and/or acts” [(52), p. 2].

It should be noted that McQuoid's research was self-published and has not been peer reviewed; however, a number of these findings have since been initially corroborated by our small, exploratory, qualitative interview study of “morally challenging behavior” among donors and recipients of online sperm donation, undertaken in 2019 (60). Three prolific, experienced donors from the UK, the US and Australia, who characterized themselves as central figures in online sperm donation, discussed their observations of, and experiences in, the online sperm donation “community”. The donors reported lying about their identity, convincing recipients that sex is more effective than artificial insemination, breaching recipients' privacy, and prejudice-based discrimination (60). Further, five recipients from the UK, Germany, Poland and Canada who had had “less than positive” experiences of online sperm donation, discussed what these experiences entailed and the impacts. The recipients reported a range of abusive behaviors occurring online and offline, such as dishonesty and deception, online harassment, sexual coercion, trolling and ghosting (60).

Although these two studies should be considered preliminary, this emerging evidence highlights that this may be a widespread problem, warranting further investigation.

By presenting the issues above, we are not necessarily suggesting that online sperm donation should, or indeed could, be regulated. The unregulated route can be of great benefit to those who choose it; but, it must be emphasized that current UK policy and practice for regulated donor insemination may be limiting individuals' opportunities to choose between the unregulated and regulated routes. Further, what this section demonstrates is that, whilst there may be advantages to the unregulated route for certain individuals, the possible implications highlight why we should not simply accept online sperm donation as a route that is commensurate with or makes up for the shortcomings in the policies and practices of, the regulated route.

## Conclusions

In this article we have considered whether restrictive policies and practices for clinical, regulated DI are a potential driver for the growing online sperm donation market. From an exploration of the NICE criteria for treatment, coupled with evidence of reduced NHS funding and inequity in its distribution, we can conclude that poor, single, LGBT+ and Black and minority ethnic women are at a significant disadvantage when attempting to access regulated DI, and even more so if they are positioned at the intersection of any number of these identities. Furthermore, from the limited research into online sperm donation, it would appear that: relationship status and sexuality are factors influencing women's decisions to look for a donor, Black and mixed ethnicity people are proportionally over-represented in online sperm donation, and lower costs are seen as an advantage of the online route. Therefore, whilst there are a range of factors driving the unregulated route to DI, it is plausible that the aforementioned policies and practices might be one of these.

We acknowledge the challenges faced by an NHS with limited resources and funds but contend that it is unjustifiable to restrict access to funded services based on an individual's sexuality or relationship status, in the same way as it is unjustifiable (and illegal) to deny treatment based on race, infertility, nationality or religion. It is also important to acknowledge the “triple jeopardy” of gender, class and race (or “quadruple jeopardy” when we also consider sexuality) discrimination experienced by minoritised individuals, who are disproportionately poor and disproportionately bear the consequences of restrictive policies and practices [(61), p. 45]. We suggest a Reproductive Justice approach to DI as a starting point for thinking about how the issues presented in this article might be addressed.

Reproductive Justice defines reproductive rights as simply: “(1) the right not to have a child; (2) the right to have a child; and (3) the right to parent children in safe and healthy environments” (51). Although the term “Reproductive Justice” was coined in the US by women of color, its goals are universally applicable because every human being should have the same human rights, with proponents emphasizing its “idealized commitment to the ‘global’” [(61), p. 6]. Reproductive Justice also places reproductive rights within the context of social and economic conditions, which shifts the focus away from the individual and onto the systemic barriers which inhibit marginalized communities from realizing their reproductive rights (62). In the case of DI services, these barriers include the restrictive policies and criteria for regulated treatment, and the subsequent withholding of state funding based on social and economic factors such as sexual orientation, relationship status, location/postcode, race/ethnicity, and insufficient income to pay for “Qualifying Treatment”. The reproductive justice approach also looks at how social and economic systems harm lives and constrain the options of the most marginalized individuals and communities. Consistent with this, we would argue that there may not be a choice between regulated and unregulated DI for single women and same-sex female couples, particularly if they are from an ethnic minority, are poor, and/or reside in areas where funding for regulated treatment has been cut (47, 60).

As per the Reproductive Justice framework, we contend that structural and systemic barriers must be interrogated and revised to enable recipients to access NHS-funded DI, if that is the route they choose. However, there are gaps in the evidence base and more definitive evidence is needed to ascertain the link between the shortcomings of current policy and the booming unregulated sperm donation market. The recent BPAS report (31) is a valuable starting point from which to conduct further research into this issue, and we suggest that a systematic review on the experiences of marginalized individuals undertaking regulated DI would help to determine how they are affected by current policies and identify any barriers they face in accessing care. It is also imperative that further research is conducted to explore the characteristics, motivations and experiences of recipients who use online sperm donation platforms, including how current policy and practice impacts on individuals' decisions and experiences. Research is currently being conducted by members of the team at Leeds Beckett University which seeks to fill these gaps. What is already evident, however, is that researching and addressing these issues will be the first steps toward more equitable provision of services and the realization of reproductive justice for *all* recipients of donor sperm.

## Data Availability Statement

The original contributions presented in the study are included in the article/supplementary material, further inquiries can be directed to the corresponding author.

## Author Contributions

FT, RT-M, and GJ led the conceptualization of the paper. FT developed the outline and drafted the paper. FT, RT-M, GJ, and AP provided comments and were involved in the writing of the paper. All authors read the final version and approved it.

## Funding

This work was supported by Leeds Beckett University as part of a part-funded Ph.D. studentship.

## Conflict of Interest

AP is the Chairman of the External Scientific Advisory Committee for Cryos (2020-2021). GJ is the Specialty Chief Editor for the Quality of Life section of Frontiers in Global Women's Health. The remaining authors declare that the research was conducted in the absence of any commercial or financial relationships that could be construed as a potential conflict of interest.

## Publisher's Note

All claims expressed in this article are solely those of the authors and do not necessarily represent those of their affiliated organizations, or those of the publisher, the editors and the reviewers. Any product that may be evaluated in this article, or claim that may be made by its manufacturer, is not guaranteed or endorsed by the publisher.
